# The Characterization of *Helicobacter pylori* DNA Associated with Ancient Human Remains Recovered from a Canadian Glacier

**DOI:** 10.1371/journal.pone.0016864

**Published:** 2011-02-16

**Authors:** Treena Swanston, Monique Haakensen, Harry Deneer, Ernest G. Walker

**Affiliations:** 1 Department of Archaeology and Anthropology, University of Saskatchewan, Saskatoon, Canada; 2 Department of Pathology and Laboratory Medicine, University of Saskatchewan, Saskatoon, Canada; University of Hyderabad, India

## Abstract

*Helicobacter pylori* is a gram-negative bacterium that colonizes the stomach of nearly half of the world's population. Genotypic characterization of *H. pylori* strains involves the analysis of virulence-associated genes, such as *vacA*, which has multiple alleles. Previous phylogenetic analyses have revealed a connection between modern *H. pylori* strains and the movement of ancient human populations. In this study, *H. pylori* DNA was amplified from the stomach tissue of the Kwäday Dän Ts'ìnchi individual. This ancient individual was recovered from the Samuel Glacier in Tatshenshini-Alsek Park, British Columbia, Canada on the traditional territory of the Champagne and Aishihik First Nations and radiocarbon dated to a timeframe of approximately AD 1670 to 1850. This is the first ancient *H. pylori* strain to be characterized with *vacA* sequence data. The Tatshenshini *H. pylori* strain has a potential hybrid *vacA* m2a/m1d middle (m) region allele and a *vacA* s2 signal (s) region allele. A *vacA* s2 allele is more commonly identified with Western strains, and this suggests that European strains were present in northwestern Canada during the ancient individual's time. Phylogenetic analysis indicated that the *vacA* m1d region of the ancient strain clusters with previously published novel Native American strains that are closely related to Asian strains. This indicates a past connection between the Kwäday Dän Ts'ìnchi individual and the ancestors who arrived in the New World thousands of years ago.

## Introduction

*Helicobacter pylori* is a helical, gram-negative, microaerophilic bacterium that inhabits the stomach of more than 50% of the world's population [1] and is one of several bacterial microbiota that are capable of colonizing the human stomach [2]. While most individuals remain asymptomatic [3], approximately 15% of *H. pylori* infections result in peptic ulcers, and 0.5% to 2% of infected individuals develop gastric adenocarcinoma [1]. There is evidence to suggest that *H. pylori* is mainly transmitted within families, especially from mother to child [4]. This infection is normally acquired in childhood, and the bacterium's genetic fingerprint remains the same for decades [5]. Although the incidence of *H. pylori* infection has decreased in geographic regions with modern sanitation infrastructures, it is still a common infection worldwide [6].

The virulence of *H. pylori* is partly determined by the type of vacuolating cytotoxin produced by the organism [7]. This cytotoxin is the result of VacA expression, and the *vacA* gene is found in all *H. pylori* strains. The *vacA* gene is generally conserved, but contains a variable middle region that may encode either an m1 or m2 allele [8]. The m1 allele has subtypes m1a, m1b, m1c and m1d [9]-[11] whereas the m2 allele has subtypes m2a and m2b [12]. The m1 and m2 alleles have been found to differ in a 300 amino acid region by approximately 50%, allowing for the differentiation of genetic variants and the determination of relatedness between bacterial strains [13]. Additionally, the m1 allele is more often linked with symptomatic disease due to the increased binding of the expressed VacA protein to host cells [3].

The *vacA* gene also contains a variable signal region consisting of either an s1 or s2 allele [8]. Subtypes s1a, s1b and s1c have been identified in s1 alleles. The s1 allele produces a fully active cytotoxin, whereas mature toxin associated with the s2 allele has an N-terminal extension that blocks vacuolation, thereby reducing toxicity. While all four possible middle and signal region allelic combinations have been reported, s2/m1 is a rare combination [3]. Strains with s1/m1 alleles produce high levels of toxin whereas little or no toxin is produced from s2/m2 alleles [14]. The s1/m1 combination is more commonly associated with peptic ulcers and gastric carcinoma [15]. A third *vacA* region, known as the intermediate (i) region, has recently been identified to have two types (i1 and i2) that are related to VacA-associated pathogenicity [16].

An additional *H. pylori* virulence factor was determined to be CagA, which is associated with the presence of the *cag* pathogenicity island (PAI) [17]. The presence of *cagA* is variable and is found in approximately 50% of the strains, and studies have shown that CagA positive strains are associated more often with severe disease [17]. Researchers discovered that CagA is injected into the host cells via a type IV secretion system that is encoded by the *cag* PAI [18].

Numerous studies have shown that genetic differences in the *H. pylori* genome are equivalent to genetic differences in human populations due to vertical transmission of the micro-organism [19]. This has led to the analysis of population movement based on genetic variation in *H. pylori*, and there is speculation that *H. pylori* has been associated with humans for thousands of years. Some of the supporting evidence includes the high levels of genetic diversity of the bacterium and the presence of similar microorganisms in non-human primates as well as many other mammals [20]. Phylogenetic analysis based on strain sequence comparisons indicate that *H. pylori* likely made the move with their anatomically modern humans hosts out of East Africa around 58,000 years ago [21].

Until recently, the only physical evidence that *H. pylori* was present in the New World prior to the arrival of Europeans consisted of the identification of *H. pylori* antigens in 3,000 year old fecal specimens [22]. In 2002, phylogenetic analyses of sequences from modern strains were incorporated into studies to determine whether *H. pylori* was indeed present prior to European contact. Yamaoka and colleagues analyzed 1,042 modern *H. pylori* isolates and identified novel *vacA* genes in eight Native Columbian and Alaskan strains. They identified that these sequences were closely related to sequences from East Asian *H. pylori* strains [11]. Additionally, gastric samples from individuals located in the Venezuelan Amazon were analyzed for evidence that *H. pylori* arrived in the New World when it was first populated by humans [23]. This study revealed that East Asian genotypes were present in all of the loci examined and suggested that *H. pylori* has been associated with humans in the New World for thousands of years. In support of this, Castillo-Rojas and colleagues identified *H. pylori* in gastric tissues obtained from a pre-Columbian male mummy approximately 50 to 60 years of age at death from the La Ventana burial cave in northern Mexico [24]. Finally, in their 2003 landmark paper on tracing human migration with *H. pylori*, Falush and colleagues noted that the *H. pylori* strains associated with the Amerind subpopulation did not indicate any signs of genetic drift as was seen in the *H. pylori* strains associated with the Maori subpopulation, where *H. pylori* likely underwent a genetic bottleneck that resulted in low genetic diversity. They commented that without evidence of drift, *H. pylori* was likely introduced into the New World in large numbers of individuals or on multiple occasions [25].

In August 1999, three hunters discovered the frozen remains of a male individual in the Samuel Glacier, which is located in Tatshenshini-Alsek Park, British Columbia, Canada on the traditional territory of the Champagne and Aishihik First Nations (CAFN). Osteological and mitochondrial evidence indicated that the ancient individual was of First Nations ancestry [26]. He was recovered with a wooden hand tool that held an iron blade, a spruce root hat and an arctic ground squirrel robe. The Aboriginal Elders named the site *Kwäday Dän Ts'ìnchi*, which means ‘long ago person found.’ An agreement was made between the provincial government and the CAFN to allow scientific analyses of the individual and the artifacts that were recovered from the site. Initial radiocarbon dating suggested that the individual was approximately 550 years old (circa AD 1450), which was a time before the arrival of Europeans [27]. However, additional radiocarbon analyses performed to clarify some original ambiguous results indicated that the Kwäday Dän Ts'ìnchi individual dated between AD 1670 and 1850, which is still precontact or early European contact in that area [28]. In this study, we report the identification and characterization of *H. pylori* DNA associated with the stomach tissue from the Kwäday Dän Ts'ìnchi individual. As *H. pylori* strains differ in virulence, regions of the virulence-associated gene *vacA* were analyzed. Also, because of the link between different *H. pylori* strains and past human migration, the *vacA* gene sequence from the Tatshenshini *H. pylori* was compared to that from modern Asian and European strains so as to add further evidence for the early presence of *H. pylori* in the New World.

## Results and Discussion

### H. pylori vacA middle (m) region

**Figure 1:**
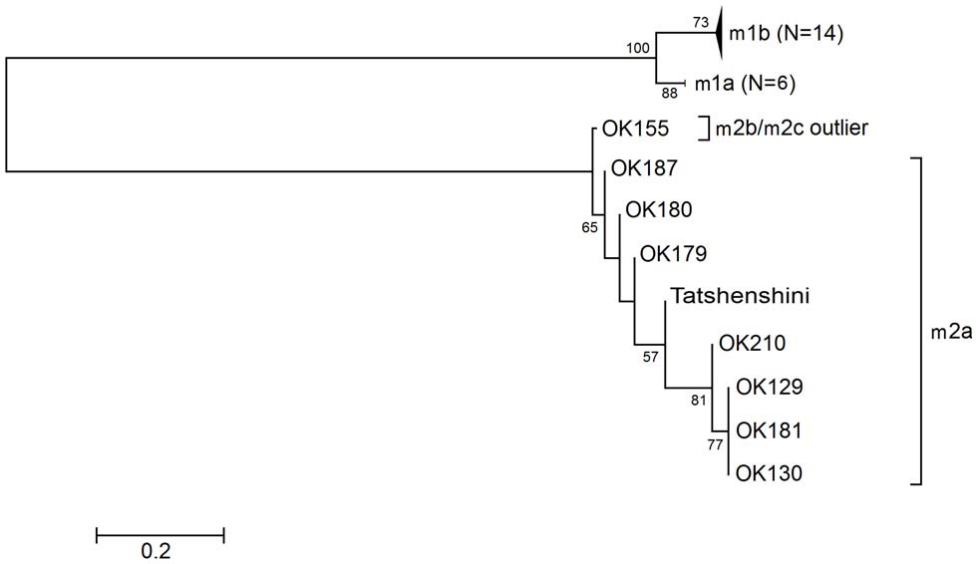
Phylogenetic tree of the portion of the *vacA* m region indicating the *H. pylori* DNA associated with the ancient stomach tissue is type *vacA* m2a. All sequence identifiers in the analysis are as described by Yamazaki and colleagues [14]. Numbers given at nodes indicate the bootstrap value as a percentage, and only values greater than 50% are displayed.

Using PCR primers MF1 and MR1 (Table 1) specifically targeting the proximal *vacA* m region of *Helicobacter pylori* [29], we successfully amplified a 180 bp fragment from stomach epithelial tissues collected from the Kwäday Dän Ts'ìnchi remains. Sequencing of this amplified fragment revealed it to be a *vacA* subtype m2a. Phylogenetic analysis indicated a close relationship between the Tatshenshini *H. pylori vacA* m region DNA sequence and *vacA* m sequences from strains isolated in Okinawa, Japan [14] (Fig. 1). Yamazaki and colleagues analyzed 220 *H. pylori* strains from the areas of Fukui and Okinawa, Japan to identify a relationship between the *vacA* gene and the clinical outcome [14]. None of the strains from Fukui had a *vacA* m2 allele, but 20 of the 105 strains from Okinawa were identified with the m2 allele. The researchers classified the *vacA* m2 alleles into a Western cluster and suggested that the appearance of the m2 allele in Okinawa was due to greater contact with the West. The similarity of the Tatshenshini *H. pylori* strain to these ‘Western’ Japanese strains in the proximal region of the *vacA* m allele suggests that Aboriginal North Americans were exposed to European *H. pylori* strains prior to AD 1850.

**Table 1:** PCR primers for the amplification of *Helicobacter pylori vacA* and *cagA* regions.

Region	Primers	Sequence (5′ – 3′)	Product size	Source
***vacA*** ** s**	VA1F	ATGGAAATACAACAAACACAC	s1 176 bp	8
	VA1XR	CCTGARACCGTTCCTACAGC	s2 203 bp	29
***vacA*** ** m**	MF1	GTGGATGCYCATACRGCTWA^a^	m1 107 bp	29
	MR1	RTGAGCTTGTTGATATTGAC^a^	m2 182 bp	29
	y98vacAmF	CCTTGGAATTATTTTGACGC	m1 479 bp	30
	y98vacAmR	ATCCATGCGGTTATTGTTGT	m2 488 bp	30
	vacAmgapF	ATGCCAGCAAGAGCGATAAT	344 bp	This study
	vacAmgapR	GCATTGTGGCCTAGGGTTAG		This study
***cagA***	cagAF	TTGACCAACAACCACAAACCGAAG	183 bp	29
	cagAR	CTTCCCTTAATTGCGAGATTCC		29
	Ako982F	ACATTTTGGCTAAATAAACGCTG	360 bp	40
	Ako9825R	TCATGCGAGCGGCGATGTG		40
	cagAnegF	GAGAGGGTGGTGCGATAAAA	236 bp	This study
	cagAnegR	GGGCTATTTTATGGGGCATT		This study

a R is A or G, W is A or T, and Y is C or T.

**Figure 2:**
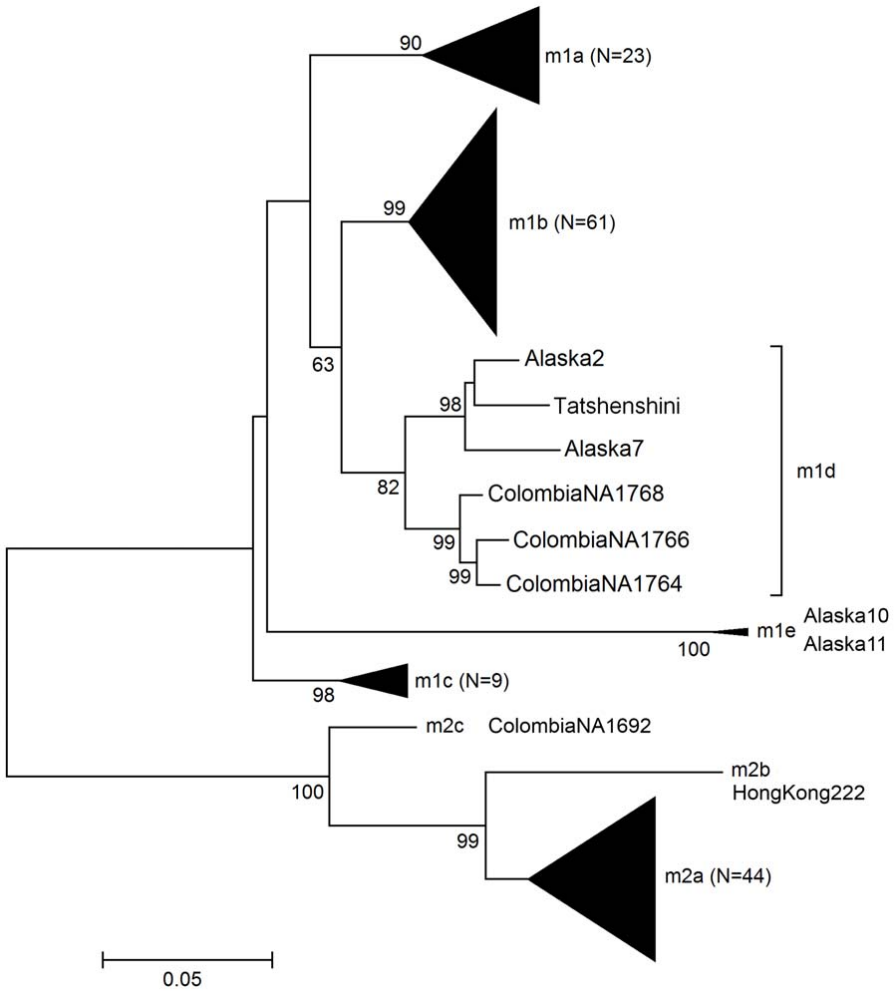
Phylogenetic tree of the *vacA* m region that indicates the *H. pylori* DNA associated with the ancient stomach tissue is type *vacA* m1d. All sequences identifiers in the analysis are as described by Yamazaki and colleagues [14] and Yamaoka and colleagues [11]. Numbers given at nodes indicate the bootstrap value as a percentage, and only values greater than 50% are displayed.

An additional 483 bp downstream of the first sequence in the *vacA* m region were amplified using the previously described primers y98vacAmF and y98vacAmR [30] (Table 1). When the Tatshenshini *vacA* m distal sequence was compared with the *vacA* m region of modern strains, the sequence clustered with sequences from m1d isolates identified in North and South American Aboriginal individuals in a study by Yamaoka and colleagues on the presence of *H. pylori* in the New World prior to Columbus [11] (Fig. 2). They identified four Native Colombian strains and four Native Alaskan strains with novel *vacA* m structures. A phylogenetic analysis indicated that five of the Native American strains (Colombia-NA1764, -NA1766, -NA1768, Alaska-2 and -7) formed a cluster that was related to the East Asian *vacA* subtype m1b. They denoted this Native American subtype as m1d [11].

Yamaoka and colleagues suggested these results indicated that Native American *H. pylori* strains did not originate with modern East Asian people, but they likely had an ancient connection, which supports the theory that *H. pylori* was associated with the first humans in the New World [11]. It is also interesting to note the connection between the strains from Alaska and the strains from Colombia. Their similarity suggests that they share a common Asian ancestor. The researchers commented that many of the Native American *H. pylori* strains have genotypes similar to those from non-Asian countries and noted that this may indicate colonization differences between Old World and New World strains [11]. The discovery of a similar *vacA* m region in the Tatshenshini *H. pylori* strain is significant because a connection has been identified between the ancient strain and local modern Alaskan strains as well as modern Asian strains.

### *H. pylori* hybrid *vacA* m region

While developing a new PCR-based typing system for untypeable *H. pylori* strains, Atherton and colleagues amplified the *vacA* m region in 77 strains from Asia, North America and South America and identified an m1/m2 hybrid allele [31]. Strain Ch2 from China was found to have an m1-like sequence at the 5′ annealing site and m2-like sequence on the 3′ end. They examined the alignments of the Ch2 sequence with the sequences from both *vacA* m1 and m2 strains, and identified a region containing the recombinational breakpoint. The Ch2 proximal *vacA* m region was type m1 whereas the distal region was type m2, which was likely the result of recombination [32]. The breakpoint for Ch2 was identified at the coordinates of 1971 bp to 1985 bp in reference strain Tx30a. Ji and colleagues sequenced the complete *vacA* gene from the Ch2 strain and identified that it is highly similar to the m1 form of the VacA protein up to amino acid 648 and similar to the m2 form from amino acid 657 onwards [33]. Vacuolation assays indicated that the chimeric toxin had the m1 phenotype, and therefore the region from amino acid 657 onwards has no impact on the phenotypic differences between strains with m1 or m2 alleles [33]. In addition, Yamaoka and colleagues analyzed the *vacA* m region of 1042 *H. pylori* isolates and identified one Japanese isolate (JapanK1) with a combination of *vacA* m1b and m2 alleles [11]. Overall, modern *vacA* m region hybrids are rare and may have arisen from a recombination between *vacA* m1 and m2 alleles during a mixed infection [34].

In this study of the Tatshenshini *H. pylori* strain, two components of the *vacA* middle region were amplified. The region that was sequenced begins at amino acid 607 based on the Shi470 strain. The proximal middle region typed as m2a whereas the distal middle region typed as m1d. At the breakpoint determined by Atherton *et al* [31], the Tatshenshini *H. pylori vacA* allele is m1. The first 27 bases of the middle region amplified using the primers from the Yamaoka *et al* study [30] at coordinate 2221 bp in the Tx30a strain is a region that is conserved between both m1 and m2 alleles. It is not possible to determine the phenotype that results from this potential hybrid m region in the Tatshenshini strain. However, a study by Pan *et al* [9] examining the association between the *vacA* genotype of modern Chinese isolates and the clinical outcome, identified four isolates with hybrid m1/m2 *vacA* alleles that contained an m1b proximal region and an m2 distal region. Higher levels of vacuolating cytotoxin were noted in these hybrid strains as compared to isolates with only an m2 allele [9]. Wang and colleagues analyzed 119 modern Taiwanese isolates and identified 104 m2 alleles, 13 m1 alleles, and two hybrid m1/m2 alleles [35]. These two hybrid alleles were highly homologous to m1 alleles in the region corresponding to bases 2701–2810 of GenBank accession number U05676, but the analysis identified a greater homology to m2 alleles in the region corresponding to bases 2540–2640 of the Tx30a isolate [35].

There is a possibility that the Kwäday Dän Ts'ìnchi individual was infected with more than one *H. pylori* strain, which would explain a *vacA* m hybrid region. Studies on modern *H. pylori* strains obtained from 65 children with and without duodenal ulcers revealed that ten children were infected with more than one *H. pylori* strain. One strain with an m1/m2 hybrid allele was identified [36]. Modern *H. pylori* isolates from 20 patients in Mexico City were characterized based on *vacA* alleles, and 17 individuals were identified as having an infection with two or more strains. Seven patients had untypeable middle regions, and five patients had s2/m1 strains. The authors concluded that co-infection with multiple *H. pylori* strains was common in Mexico, and these strains appeared to have more diversity than those associated with other populations [37]. Also, a study by Kim and colleagues showed that the genotypes of *H. pylori* isolates differed from the biopsy genotypes from the same individual [38]. Unfortunately, the complete *vacA* m region of the Tatshenshini *H. pylori* strain could not be amplified. A breakpoint was not determined, but the strain was potentially a true *vacA* m hybrid as opposed to a dual or mixed infection because of the clean forward and reverse sequencing runs of the two *vacA* m regions.

### *H. pylori vacA* signal (s) region

Previously published primers were used to amplify 203 bp in the *vacA* s region (Table 1). The Tatshenshini *H. pylori* strain contained the *vacA* s2 allele with a type A signal region insert. Previous studies have shown that toxigenic *vacA* type s1 strains encode a protein that has a hydrophobic N-terminal region that can insert into lipid bilayers, whereas this region in non-toxigenic s2 strains contains a hydrophilic N-terminal extension that blocks vacuolating activity [7]. Interestingly, studies have shown that s2/m2 VacA is capable of vacuolating activity if the N-terminal extension is removed [7]. It is not understood why some *H. pylori* strains have a blocked capability of vacuolation.

Phylogenetic analysis indicated a close genetic relationship between the *vacA* s allele of the Tatshenshini *H. pylori* strain and the *vacA* s alleles from modern s2 strains isolated from North and South American Aboriginal individuals [11]. Modern isolates were analyzed by Yamaoka and colleagues for a study on the presence of *H. pylori* in the New World before Columbus [11]. They characterized 1042 isolates based on variables including the *vacA* genotype. Most of the East Asian *H. pylori* strains were *vacA* s1c (94.7%), and none of the strains were s2. They identified novel Native American *H. pylori* strains with an s1 allele. Some of the strains (Colombia-NA1692, Alaska-2 and Alaska-7) clustered close to s1c, yet phylogenetic analysis of the *vacA* s region indicated that sequence differences between established subtypes were very low (for example between s1c and s2) [11]. The Tatshenshini s2 sequence is highly similar to the *vacA* s sequences of two modern Alaskan *H. pylori* strains, Alaska-8 and -14 in this study. Interestingly, these Alaskan strains were typed as m2a in the *vacA* m region with sequences similar to the m2a region in the Tatshenshini *H. pylori* strain. It is noteworthy to mention that the Kwäday Dän Ts'ìnchi site was discovered less than 50 km from the British Columbia-Alaska border, which is intriguing considering the phylogenetic analysis revealed that two modern Alaskan strains share a high percentage identity in the *vacA* s region with the strain associated with the Kwäday Dän Ts'ìnchi individual.

The s2 subtype is not associated with Asian strains. *H. pylori* isolates were analyzed by Ghose and colleagues from patients located in Caracas, Venezuela and a center in the Venezuelan Amazon known as Puerto Ayacucho [23]. The individuals from Caracas had European or mixed ancestry whereas the individuals from Puerto Ayacucho were of Amerindian ancestry. The isolates from Caracas were identified with either *vacA* s1b or s2 alleles whereas half of the Puerto Ayacucho isolates had *vacA* s1c alleles, which has been identified in East Asian strains [23]. Interestingly, 1 of the 17 Puerto Ayacucho isolates was characterized with an s2 allele. These findings suggest that non-indigenous genes may have been introduced into Puerto Ayacucho. The identification of an s2 allele in the Tatshenshini *H. pylori* strain suggests that European strains were present in northern British Columbia prior to his lifetime.

Inserts within the s region of the *vacA* gene were analyzed in a previous study that involved 484 modern isolates from 32 countries [39]. The short inserts (27 bp) found in s2 strains were highly conserved, and no connection with geographic origin was identified. Even with numerous DNA polymorphisms, most (98%) of the s region inserts contained the NDPIHSESR amino acid sequence [39]. The analysis of the Tatshenshini *H. pylori* strain revealed that the same conserved amino acid sequence was present. The previous study also identified that most s2 sequences contained a pre-insert motif (MGTELGANTP) in the s region (SRP type I) before the insert site. Five other SRP types were defined including M (1) to I or G (2) to S substitutions found in 10% of the strains [39]. This study identified that the Tatshenshini *H. pylori* s region contained a pre-insert amino acid motif IGTELGANTP.

### H. pylori cagA

In this study, the *cagA* status of the Tatshenshini *H. pylori* strain was not determined due to lack of amplification in that region. Unfortunately, no *cagA* PCR products were obtained when previously published primers and primers specifically developed for this study were used [29], [40]. Since a false negative result may be due to DNA degradation, further steps were taken to determine if the region around the *cagA* PAI could be identified. The *cag* PAI is flanked by 39 bp direct DNA repeats, and *H. pylori* strains that are *cagA* negative do not have a complete PAI, but they do possess a single copy of the 39 bp sequence in the glutamate racemase gene. Partial *cag* islands and size variation have been identified [41]. Unfortunately, we were unable to confirm that the *H. pylori* strain was *cagA* negative because no DNA amplification occurred with primers that flanked the direct repeat region.

### Conclusion

In this study, *H. pylori* DNA was amplified and sequenced from the stomach tissue of the approximately 200–300 year old Kwäday Dän Ts'ìnchi remains recovered from the Samuel Glacier in Tatshenshini-Alsek Park, British Columbia, Canada. It is significant to find evidence of this ancient pathogen because other than a recent publication by Castillo-Rojas and colleagues [24], only modern *H. pylori* strains have been studied. While phylogenetic analyses suggested that this bacterial pathogen has been present throughout human history, the antiquity of this bacterium is confirmed through the study of *H. pylori* DNA recovered from an archaeological context. Naturally mummified individuals are more suitable for bacterial DNA studies because they have not been impacted by any processes such as embalming that would alter the tissue environment [42]. The confirmation of the presence of bacterial pathogens associated with ancient individuals is an important part of determining the temporal extent of infections affecting humans.

Through an analysis of the Tatshenshini *H. pylori vacA* gene, a potential hybrid m2a/m1d allele and an s2 signal region allele were identified. The presence of a *vacA* s2 allele, which is unusual in Asian strains, suggests that European *H. pylori* strains were present in the region during the timeframe of AD 1670 to 1850. The characterization of the *vacA* m region revealed a potential hybrid region that is rare in modern strains. The phylogenetic analysis indicated that the m1d sequence clustered with previously studied sequences from novel Native American strains that are closely related to Asian strains. These observations are consistent with the idea that the first humans who migrated into the New World crossed over the Bering Strait from Asia.

In 2006, Canadian Aboriginal communities were identified by a Canadian *Helicobacter* Study Group as a population at most risk of developing a *Helicobacter*-related disease [6]. Studies in the circumpolar region also identified high levels of *H. pylori* infections in the communities of Greenland and Russia. Based on the identification of the potential *vacA* hybrid m region in the ancient Tatshenshini *H. pylori* strain (*vacA* m2a/m1d), this suggests that due to the current high rates of infection in the circumpolar region, further studies need to include the identification of complete *vacA* m sequences in the modern *H. pylori* strains isolated from individuals living in Northern communities to gain a better understanding of the role that the *vacA* m region plays in the virulence of the bacterium in that locality.

## Materials and Methods

### Sample Collection

Approximately 0.7 grams of stomach epithelial tissue was dissected from the Kwäday Dän Ts'ìnchi individual at the Royal British Columbia Museum in Victoria, Canada during the retrieval of a variety of other tissue samples for use by multiple research teams. Standard protocols were followed to prevent contamination of the samples. All members of the autopsy team were dressed in appropriate protective clothing, and sterile surgical tools were used during sample collection. The stomach samples were frozen and packed on ice in an insulated container for travel to the University of Saskatchewan, in Saskatoon, Saskatchewan, where they were stored in a −70°C freezer upon arrival.

### DNA Extraction

Tissue extractions were performed in a biological safety cabinet that was surface cleaned with 10% (v/v) Clorox® bleach. A sterile scalpel was used to mince 0.24 grams of stomach tissue into small fragments, and the DNA was extracted using the tissue protocol with the QIAamp® DNA Mini Kit (QIAGEN Inc., Mississauga, Ontario).

### PCR Amplification and Electrophoresis

All PCR reactions were set-up in a separate location from the post-PCR laboratory. The components of the 50 µl reaction consisted of: 5 µl DNA extract, 2 Units AmpliTaq® Gold DNA Polymerase (Applied Biosystems Canada, Streetsville, Ontario), GeneAmp® PCR Gold Buffer (15 mM Tris-HCl, pH 8.0 and 50 mM KCl), 2.5 mM MgCl_2_, and 200 µM each dNTP from GeneAmp® dNTP Mix (Applied Biosystems Canada, Streetsville, Ontario). Previously published and newly designed PCR primers (Sigma Genosys Canada, Ontario) were used for the amplification of the *Helicobacter pylori vacA* variable regions (Table 1). PCR reactions were performed in an MJ Mini Gradient Thermal Cycler (Bio-Rad Laboratories, Hercules, California) using amplification conditions as previously described [29], [30] or as follows when using the newly designed vacAmgap primers: 12 minutes initially at 95°C, followed by 45 cycles of denaturation at 95°C for 1 min, annealing at 52°C for 1 min, extension at 72°C for 1 min, and completed with a 72°C incubation for 8 minutes. Following amplification, five microlitres of the reaction products were electrophoresed through 2% (w/v) agarose gels in a Tris/acetate/EDTA buffer solution. Following electrophoresis, gels were stained with 0.5 µg/ml ethidium bromide, destained with water, and visualized with ultraviolet light.

### DNA Sequencing and Phylogenetic Analysis

PCR products were sequenced in both directions on an ABI 3730*xl* DNA sequence analyzer at the National Research Council-Plant Biotechnology Institute (Saskatoon, Saskatchewan). The sequences in this paper have been deposited in the National Institute of Health (NIH) GenBank database with the accession no. HM778162. They were compared with reference sequences in the GenBank database, and phylogenetic analyses were used for the determination of evolutionary relationships. Sequences were aligned with the ClustalX software program [43] and DNA alignments were subsequently visualized and manually edited using the GeneDoc software program [44]. All phylogenetic trees were produced and visualized with the Neighbor-Joining algorithm using MEGA4 (Molecular Evolutionary Genetics Analysis software version 4.0) [45]. Tree topology was also evaluated using Minimum Evolution, Maximum Parsimony and Unweighted Pair Group Method of Arithmetic Means algorithms and found to produce a similar overall topology to that of the Neighbor-Joining method. A bootstrap test [46] of 1000 replicates was performed.
